# Analysis of Toxic Metals in Aerosols from Devices Associated with Electronic Cigarette, or Vaping, Product Use Associated Lung Injury

**DOI:** 10.3390/toxics9100240

**Published:** 2021-09-29

**Authors:** Nathalie Gonzalez-Jimenez, Naudia Gray, R. Steven Pappas, Mary Halstead, Erica Lewis, Liza Valentin-Blasini, Clifford Watson, Benjamin Blount

**Affiliations:** Tobacco and Volatiles Branch, National Center for Environmental Health, Centers for Disease Control and Prevention, 4770 Buford Hwy NE, MS S110-4, Atlanta, GA 30341-3717, USA; NGonzalezjimenez@cdc.gov (N.G.-J.); NRGray@cdc.gov (N.G.); MHalstead@cdc.gov (M.H.); ELewis1@cdc.gov (E.L.); LValentin@cdc.gov (L.V.-B.); CWatson@cdc.gov (C.W.); BBlount@cdc.gov (B.B.)

**Keywords:** cannabis, vaping, vape, aerosol, toxic, metals, lung, EVALI

## Abstract

Research gaps exist in toxic metals characterization in e-cigarette, or vaping, products (EVPs) as these analytes typically have low concentrations and most standard aerosol trapping techniques have high metals background. An additional complication arises from differences in the EVP liquid formulations with nicotine products having polar properties and non-nicotine products often being non-polar. Differences in polar and non-polar matrices and the subsequent aerosol chemistries from various EVPs required modifications of our previously reported nicotine-based EVP aerosol method. Validation and application of the expanded method, suitable for both hydrophobic and hydrophilic aerosols, are reported here. The metals analyzed for this study were Al, Cr, Fe, Co, Ni, Cu, Cd, Sn, Ba, and Pb. The method limits of detection for the modified method ranged from 0.120 ng/10 puffs for Cd to 29.3 ng/10 puffs for Al and were higher than reported for the previous method. Results of the analyses for metals in aerosols obtained from 50 EVP products are reported. Cannabinoid based EVP aerosols were below reportable levels, except for one sample with 16.08 ng/10 puffs for Cu. Nicotine-based EVP results ranged from 6.72 ng/10 puffs for Pb to 203 ng/10 puffs for Sn. Results of the analyses for these metals showed that aerosols from only 5 of the 50 devices tested had detectable metal concentrations. Concentrations of toxic elements in the aerosols for nicotine-based EVP aerosol metal concentration ranges were consistent with previously published results of aerosol analyses from this class of devices.

## 1. Introduction

According to the National Youth Tobacco Survey and epidemiological surveillance published by the Centers for Disease Control and Prevention, e-cigarettes are the most commonly used tobacco product among U.S. youth between 2019 and 2020 [1]. The use of EVPs, however, remains the most common means of nicotine consumption among youth and young adults in the U.S. [2,3]. The highest prevalence of EVP use among adults is in the age range 18–24 [4]. Between 2017 and 2019, EVPs became increasingly popular means of both nicotine and cannabinoid delivery among teens and young adults [5,6]. The use of cannabis product vaping devices increased between 2018 and 2019 from 11% to 14% of U.S. college students and from 8% to 17% of the students’ age-matched non-student peers [6]. The growing popularity of vaping as a means of delivery of both nicotine and cannabinoids has increased the need for the development of well validated methods for the analysis of potentially harmful substances such as toxic metals in EVP liquids [7]. Rigorous and validated analytical methods are also needed for measuring harmful substances including toxic metals in the aerosols generated by EVPs [8,9] so that inhaled exposure can be assessed. Furthermore, concurrent and/or dual use of both nicotine and cannabinoid EVP liquids is increasing, and thus robust methods are needed for diverse EVP liquids and aerosols [10].

A health emergency was recently declared in the United States involving vaping devices. Initially, the etiologic agent or agents causing the E-cigarette, or Vaping, Product Use-Associated Lung Injury (EVALI) epidemic were unknown. Analyses for a broad spectrum of substances in bronchoalveolar lavage fluid (BALF) and in product aerosols including toxic metals were performed during the investigation. Analyses of BALF from hospitalized patients showed the presence of inhaled hydrophobic substances, most commonly, vitamin E acetate [11].

A recent study reported that elevated levels of metals such as Ni, Cu, Zn, and Pb are transported in EVP aerosols and that the heating elements were the principal sources of the metals [12]. Studies using methods with well determined limits of detection also indicated that the corrosive nature of the liquid toward metal device components in contact with the liquids as well as the ages of the devices were important factors that contributed to liquid and aerosol metal concentrations [7,8]. Studies of aerosols using single particle and dual element single particle inductively coupled plasma-mass spectrometry (SP-ICP-MS) provided evidence that electrical connectors and other device components, other than the heating element, were the more likely sources of chromium or nickel containing particles [13].

Metal concentration ranges have not been well characterized for aerosols from EVP devices containing cannabinoids. One study reported silicon, copper, nickel, and lead at concentrations up to 600 ppm in liquids from devices obtained during the 2019 e-cigarette, or vaping, product use-associated lung injury (EVALI) response [14]. This information is valuable, but information on metal concentrations in aerosols that are inhaled from these devices would be a valuable exposure predictor. Although cobalt has not been reported as a significant constituent of any components of vaping devices, the liquid from an EVP vaping device containing cannabinoids used by a patient (who had formerly smoked tobacco) was analyzed after the patient was diagnosed with giant cell interstitial pneumonia secondary to cobalt exposure [15]. The authors stated that cobalt (but not tungsten) was found in the EVP liquid analyzed according to the method of Hess et al. [16], although several of the elements in the analytical results in the report [15], including cobalt and tungsten, were not included in the cited method described by Hess et al. [16]. Neither specifics of the method, nor limits of detection were described in either the authors’ report nor the cited method [15,16]. Although the authors affirmed that cobalt was found in the EVP liquid, neither cobalt nor tungsten containing particles were found in lung tissue samples from the patient [15]. Reports that leave unanswered analytical questions such as these support the need for data obtained using validated methodology.

The analysis of hydrophilic EVP aerosols for toxic metals can be challenging, since aerosols that contain very low metal concentrations must be trapped using materials that do not leach metals into the samples to avoid sample contamination [8,9,10]. Common solvents in nicotine containing EVP are propylene glycol and glycerol which make them hydrophilic [17,18]. Sample preparation for these hydrophilic aerosols may be as simple as dilution of the aerosol in high purity aqueous acids [8,9]. However, the analysis of EVPs with hydrophobic aerosols is more challenging. The use of glass vessels or digestion tubes must be avoided as a part of good inorganic analytical practice, since glass vessels and tubes are not metal free, and leaching glass vessels with acid does not eliminate extractable metals [10]. The analysis of toxic metals in EVP with hydrophobic aerosols is also more complicated due to the greater variety of solvents used in these devices for cannabinoid delivery. EVP devices can have a either hydrophilic or hydrophobic solvents [17,18] and some EVP devices with hydrophobic solvents can contain cannabinoids [10]. Common hydrophobic solvents that are, or have been, used in EVP devices are vitamin E acetate and medium-chain triglyceride (MCT) oil. Vitamin E acetate is a hydrophobic oil soluble antioxidant vitamin derivative that is safe when ingested but was not intended to be inhaled. MCT oil comes from oil sources such as palm oil or coconut oil. Neither MCT oil nor vitamin E acetate is miscible with water, so a solvent that will dissolve hydrophobic substances was required to remove the aerosols from the aerosol traps [10].

The method described here is a modification of our previously published method with a high purity fluoropolymer tube used for trapping aerosol from EVP nicotine based devices [8] that has been adapted with an additional solvent for recovery of hydrophobic and hydrophilic substances in aerosols. The quantitative method employed triple quadrupole inductively coupled plasma mass spectrometry (QQQ-ICP-MS) for analysis of aluminum, chromium, iron, cobalt, nickel, copper, cadmium, tin, barium, and lead in a subset of 50 case related EVPs, obtained during the CDC 2019 U.S. EVALI response [11].

## 2. Materials and Methods

### 2.1. Vaping Procedures

A CETI-8 e-cigarette vaping instrument (Cerulean, Richmond, VA, USA) was used for aerosol generation. Before each run, puff volumes were verified using a calibrated soap bubble meter. CORESTA Method 81 parameters were used for aerosol collection (3 s 55 mL puff every 30 s with a rectangular puff profile, a machine pressure drop ≤ 300 Pa and a pressure drop for the aerosol trapping assembly ≤ 900 Pa at an air velocity of 140 mm/s) [19]. Due to the limited volume of liquid remaining in many devices as received, aerosol from 15 puffs was generated for each sample.

### 2.2. Aerosol Collection

The aerosol capture procedure used for EVP nicotine based devices [8] was modified to accommodate hydrophobic aerosol generated from EVP devices. Prior to use and between uses, the 518 cm, 3.97 mm i.d. fluorinated ethylene propylene (FEP) tubing (Savillex, Eden Prairie, MN, USA) was cleaned repeatedly with puriss. p.a. acetone obtained in high density polyethylene (HDPE) bottles (Sigma, St. Louis, MO, USA) and 2% *v*/*v* nitric acid + 1% *v*/*v* hydrochloric acid and dried under vacuum. The tubing was connected tightly to the mouthpieces of the EVP devices with acid cleaned tygon tubing. The other end of the tubing was connected to the vaping machine syringe pump with an in-line polytetrafluoroethylene (PTFE) 60 mm filter with 0.45 µm pore size to prevent late condensing aerosol from accumulating in the vaping machine syringe pump. The aerosol generated was trapped by condensation inside the FEP tubing with 64.1 mL internal volume, approximately 17% greater than the puff volume. Aerosol recovery in the condensation tubing trap was calculated as the mean % of three replicate recoveries using 80% vitamin E acetate, 20% hemp oil aerosol mass recovered in the tubing and the post-tubing filter.

### 2.3. Aerosol Sample Capture Procedures

Appropriate solvent selection and executing good analytical practices were a critical part of the development of this method. The requirement for a nonaqueous organic solvent presented additional challenges for inorganic analysis including purification of the solvent for metals analysis and how to proceed after removing the sample in an organic solvent from the trap. In order to avoid an additional drying or sample digestion step, a high purity solvent that dissolves hydrophobic substances, that are also water miscible was required. Therefore, diethylene glycol monoethyl ether (DEGMEE) was chosen. This solvent was used to rinse both hydrophobic and hydrophilic aerosols from the high purity FEP collection trap. Currently, no ultrapure grade of DEGMEE is available for trace metals analysis; therefore, the DEGMEE was distilled using a high-purity fused silica quartz distillation flask prior to use. The aerosols generated by devices condensed inside the FEP tubing traps and were flushed using initial rinse with 5 mL of quartz distilled DEGMEE (Sigma Aldrich, St. Louis, MO, USA), followed by 4 × 8 mL rinses with 2% *v*/*v* nitric acid (Environmental grade, GFS, Powell, OH, USA) further purified in a PFA sub-boiling still (CEM, Matthews, NC, USA), and 1% *v*/*v* hydrochloric acid (Veritas double distilled, GFS, Columbus, OH, USA). The 4 × 8 mL tube acid rinses were added to the 5 mL DEGMEE rinse in 50 mL acid cleaned polymethylpentene class A volumetric flasks. Samples were brought to 50 mL using the same 2% *v*/*v* nitric acid + 1% *v*/*v* hydrochloric acid rinsing solution then transferred to acid cleaned 50 mL polypropylene (PP) sample tubes. PP tubes from two vendors were tested for leachable metal backgrounds. Higher aluminum and iron backgrounds were leached from one vendor’s tubes even after prerinsing the tubes with 1% *v*/*v* nitric acid. The tubes that contributed lower metal concentrations to blanks, Falcon (New York, NY, USA), were chosen as sample containers. Procedural blanks were prepared in these tubes after using the fluoropolymer trap and the same rinsing and sample preparation procedure as the samples.

### 2.4. Analysis of the Samples

Calibration standards were prepared in 2% *v*/*v* nitric acid, 1% *v*/*v* hydrochloric acid, 0.25% *v*/*v* hydrofluoric (Veritas double distilled, GFS), and 10% DEGMEE by diluting NIST traceable single element standards obtained from High Purity Standards (Charleston, SC, USA). The calibration blanks were the acid solution used to prepare calibration standards. The calibration ranges for five standards were 0.040 to 0.500 µg/L cadmium, 0.080 to 1.00 µg/L tin, 0.200 to 2.50 µg/L chromium, cobalt, nickel, barium, and lead, 0.400 to 5.00 µg/L copper, 1.00 to 12.5 µg/L iron, and 4.00 to 50.0 µg/L aluminum. Sample concentration results in ng were calculated by multiplying calibrated results in µg/L by the final analytical volume (0.050 L) and by 1000 ng/µg to convert µg to ng of metal transported. Sample results in ng obtained from 15 puffs were then divided by 1.5 to express concentrations in terms of ng per 10 puffs.

Two analytical quality control (QC) solutions were prepared with 400 µL of medium-chain triglyceride (MTC) Oil (Prasada, Portland, OR, USA) for hydrophobic matrix approximation. Second source single element standards (Inorganic Ventures, Christiansburg, VA, USA) were diluted to 50.0 mL with the MCT oil, 2% *v*/*v* nitric acid, 1% *v*/*v* hydrochloric acid, and 10% *v*/*v* DEGMEE. Duplicate low and high QCs were analyzed before and after samples during each analytical run. Results from the aerosol procedural blank were subtracted from QC and sample concentrations in each analytical run. Quality control was maintained using a modified Westgard approach [20] using SAS software (Cary, NC, USA). If a QC failed, the run was repeated if a sufficient sample remained. When insufficient inventory of any sample remained, analyses could not be repeated, and results for which QCs had failed were not considered reportable.

Liquid samples were analyzed for nicotine and cannabinoids such as Δ9-tetrahydrocannabinol and cannabidiol using methods to be reported separately to verify the purpose of the devices used in this study. If cannabinoids were detected, the devices were considered as cannabinoid devices whether nicotine was also detected in devices that were repurposed for generating aerosol containing cannabinoids.

### 2.5. Instrument Parameters

The EVPs diluted aerosol samples were analyzed using an Agilent (Tokyo, Japan) 8800 QQQ-ICP-MS with an Elemental Scientific (Omaha, NE, USA) SC4-DX FAST autosampler. Samples were taken up using 0.38 mm peristaltic pump tubing with a pump speed of 0.42 rps. Samples were diluted 1:1 using tubing of the same diameter teed in with internal standard solution (1.0 µg/L rhodium and 2.0 µg/L thulium in 1% *v*/*v* nitric acid and 1% *v*/*v* hydrofluoric acid). Diluted samples were introduced into the plasma after desolvation using an Elemental Scientific Apex introduction system and C400 concentric PFA nebulizer (Savillex, Minnetonka, MN, USA). The plasma was maintained at 1550 watts RF power, 15 L/min plasma gas, and 0.90 L/min auxiliary gas, optimized near 5.8 mm sampling depth for low oxides. Nebulizer gas was optimized as needed for signal intensity and stability while maintaining oxide formation below 1%. Lens parameters were optimized as needed except for method and mode-specific parameters described in Table 1.

### 2.6. Scanning Electron Microscopy (SEM-EDS)

Components of an atomizer from a representative Jupiter CCELL^®^ Liquid6 510 Thread Cartridge glass tank system with unknown heating element voltage (https://www.jupiterresearch.com) (accessed on 21 September 2021) were analyzed using an FEI Quanta 250 Field Emission instrument (Hillsboro, OR, USA) with Oxford Energy Dispersive X-ray system and 80 cm^2^ Silicon Drift Detector (SEM-EDS). The SEM analysis was obtained at 20.00 kV beam energy using an Everhart Thornley Detector in High Vacuum mode at 7.92 × 10^−5^ Pa.

### 2.7. EVALI Application

Single replicate analyses of 15 puffs obtained from each of the 50 devices from 2019 EVALI case patients were analyzed in 2020 as part of the CDC EVALI response. The products were collected from federal and state partners. Samples arriving at the CDC were handled using the chain of custody protocols. Samples were vaped using fully charged and compatible batteries. If a working, compatible battery was included in the same case study as a cartridge or pod, then that battery was used for the corresponding sample. The mass of the entire device was recorded before and after the vaping session. Only runs with adequate aerosol production, defined in this study as >5 mg lost in the product during aerosol generation from 15 puffs, were considered for reporting.

### 2.8. Validation

Accuracy was determined by spiking low, mid, and high levels of all analytes across the calibration range along with 400 µL of MCT oil diluted to 50 mL with 2% *v*/*v* nitric acid, 1% *v*/*v* hydrochloric acid, and 10% *v*/*v* DEGMEE. The solutions were spiked using second source Inorganic Ventures standards compared with the High Purity Standards calibration for quantification. The accuracy results ranged from 91% to 106%. Accuracy results are shown in Table 2 and Table 3. Calibration curve linearity was confirmed by residual analysis of the linear regression of seven calibration curves with a coefficient of determination, r2, of ≥0.98.

Method precision was determined by assessment of results variability duplicate quality control samples from 8 analytical runs over a period of 8 days. Repeatability was calculated as a within-run variation of duplicates, while intermediate precision was calculated as a between-run variation (Table 4). The method limits of detection (LODs) were calculated according to Taylor [21], with standard deviations of the five calibration standards, the procedural blank, and the low and high QC results from 8 analytical runs plotted against concentrations with regression lines extrapolated to S_0_. S_0_ was multiplied by 3 to determine the preliminary method LOD. The final LODs were statistically adjusted higher to avoid random overlaps between false positives, and false negatives [22]. Method LOD calculations were validated by repetitive analyses of analytes spiked at the LOD and at two concentrations above LOD. If the results obtained from analysis of the spikes at the calculated LOD for any analyte were not accurate then the LOD was adjusted to the higher to the spike concentration at which accuracy was demonstrated (Table 3). The lowest reported level (LRL) was the respective lowest standard concentration for each analyte expressed in terms of ng aerosol or the method LOD, whichever was the higher of the two. Method limits of detection and lowest standard concentrations are shown in Table 5.

## 3. Results

The components of an atomizer from a representative glass tank cannabinoid delivery system were evaluated by SEM/EDS. The EDS analysis indicated that the ceramic heating block was composed primarily of aluminum and silicon with only superficial sodium. The wires connecting the heating block to the battery were composed of Ni and Cr and the holder was primarily composed of Ni. These materials in this cannabinoid delivery system were similar to those previously reported for nicotine delivery systems, although nickel was more prevalent than copper and brass [7,8,9].

Aerosol recovery in the condensation tubing was 87 ± 3%, the majority of the aerosol mass, as was the case when the same condensation tubing trap was used for hydrophilic aerosol collection [8].

Method validation results including aqueous matrix and oil matrix spike accuracies, method precision and limits of detection are shown in Table 2, Table 3, Table 4 and Table 5, respectively. Comparison of spike recovery accuracies in aqueous and oil matrices negligibly different and ranged from 91% for the nickel low spike to 105% for the iron high spike in aqueous blanks, compared to a range from 93% for the barium low spike to 106% for the nickel low spike in oil.

For this study, a combination of EVP nicotine- and cannabinoid-based samples that were obtained from EVALI patients were analyzed. The aerosol results were reported per 10 puffs (55 mL/puff), which serves as a basis for comparison to cigarette aerosol deliveries since 10 puffs is in the intermediate range of puffs for US cigarettes smoked using the World Health Organization intense smoking regimen [23]. For most samples, metal concentrations were <LOD. Of the 50 samples analyzed, only five samples had reported levels for toxic metals and are shown in Table 6.

Reportable levels of Cu in four different samples were 73.15 ng/10 puffs, 447.15 ng/10 puffs, 15.4 ng/10 puffs, and 16.06 ng/10 puffs. Two samples had levels of Ni with 15.5 ng/10 puffs and 36.0 ng/10 puffs. For Sn, two samples had reportable levels with 4.77 ng/10 puffs and 202 ng/10 puffs. Only one sample had reportable revels for Pb with 6.71 ng/10 puffs. Because available sample volume only permitted one analytical replicate, results that were slightly above our reportable level, could not be repeated. It should be noted that method LODs reported for this study were slightly higher than reported in a previous study [9] as a result of the analysis of only 15 puffs of aerosol due to limited availability of sample volume and possibly due to the additional tube rinse with an organic solvent.

A subset of the 50 samples, 45 samples had matching results from separate analyses using appropriate methods for product categorization of delta-9-tetrahydrocannabinol (9-THC), cannabidiol (CBD), and nicotine. The samples were categorized based on active ingredient concentrations: Of the 45 samples, 39 were positive for THC, CBD, or both, and 11 samples were positive for nicotine content. One sample was negative for all categories. These results were used for categorization of products in Table 6. Sample 5 from Table 5 is the only sample with THC and CBD content with reportable levels of metals with a concentration of 16.1 ng/10 puffs Cu.

For the 11 nicotine products, four samples (Sample 1-Sample 4 shown in Table 5) resulted in reportable levels for metals. These four samples came from pod-type devices. The highest reported metal concentrations were Cu with 447 ng/10 puffs and Sn with 202 ng/10 puffs. The values for the nicotine samples are similar to previous reported results (Cr, Ni, Cu, Cd, Sn, and Pb also included in previous study) in nicotine based EVP pod-type devices utilizing the hydrophilic aerosol analysis approach [8]. In the previous study, metal concentrations in aerosols ranged from below that method’s lowest reportable levels to 29.9 ng/10 puffs for Cr, 373 ng/10 puffs for Ni, 209 ng/10 puffs for Cu, 127 ng/10 puffs for Sn and 463 ng/10 puffs for Pb [9]. All samples were below reportable levels for cadmium in both studies [9].

## 4. Discussion

The method has proven to be suitable for both hydrophilic and hydrophobic aerosols. Our results for hydrophilic aerosols compare well with previously published results. Overall, there was a difference between the results for cannabinoid-based EVPs (all 10 metals except Cu for one sample below the lowest reportable levels) and nicotine-based EVPs (some metals well above the lowest reportable levels). The aerosol produced from the cannabinoid-based EVPs differed from nicotine-based EVPs with the product mass lost averaging 26.5 ± 18.1 mg and 68.9 ± 34.8 mg respectively. Although there was variability between the different samples, in general, more aerosol was produced by the nicotine-based EVPs. More aerosol could result in higher metal inhalation by users. However, some of the metals in nicotine-based EVPs were well above our detection limits. For example, Sample 2 Sn concentration (202 ng/10 puffs) is almost 100 times larger than the lowest standard (2.67 ng/10 puffs). Limitations of the data reported here include the single replicate analyses and the small number of puffs obtained due to limited sample quantity. A greater dilution factor was also required for cannabinoid-based EVPs due to the organic solvent, which contributed to higher method LODs.

Metals in nicotine-based EVP aerosols have been shown to be correlated with the metal components in contact with the device liquids [8,9]. The differences between the physical characteristics of the active ingredients present in the aerosol from nicotine-based EVPs and cannabinoid-based devices can be because of the liquid composition and the differences in the product design. The cannabinoid-based EVPs analyzed in this study all resembled ceramic cell cartridge technology and used a ceramic heating element. This ceramic heating element may provide more surface area for aerosol production from the more viscous liquids used in cannabinoid-based devices [24]. Cannabinoid-based EVP devices also contain a heating element and connectors, but they are more insulated from the liquid by the ceramic fiber wicking [25]. The design of the nicotine-based EVPs often consists of a silica wick which can absorb the less viscous propylene glycol and glycerol solvents. For the types of pods in this study, the wicking components were silica wicks or porous ceramic blocks [9]. The alloy heating elements and electrical contacts that are all in direct contact with the liquid in the pods are contributors to aerosol metals [9]. The differences in liquid composition and device design are contributing factors in metal concentrations in the EVP device nicotine based and cannabinoid-based aerosols. In this study, the products were not purchased directly from manufacturers and brands are not known. Therefore, the conclusions about internal component designs are from visual observations of the samples.

## 5. Conclusions

EVPs with different solvent systems were used to generate aerosol samples and analyzed alongside each other using our new method. We found this approach to be suitable for accurate analyses of hydrophobic aerosol and for hydrophilic aerosols. Clean sample preparation including avoidance of glass was important for maintaining low metals backgrounds. The results obtained from the analysis of nicotine-based EVP aerosols in this study were similar to results reported in a previous study [9]. Metal concentrations in cannabinoid-based EVP aerosols analyzed in this study were almost entirely below reportable levels using a validated method. The samples analyzed represent a “convenience” sampling and should not be construed as being representative of the emerging EVP market. As EVP product designs continue evolving it is important to continue to monitor emissions of both nicotine-based and cannabinoid-based devices. In this study we used the standardized CORESTA Recommended Method 81 puff regimen for aerosol collection as a mean to yield reproducible data; however, users of these products can inhale much more diverse and broader amounts of aerosol.

Metals exposure from aerosols originating from nicotine or cannabinoid based EVPs aerosols could have serious health effects such as pulmonary inflammation, sensitization, toxicities, and cancer [26]. Because of the increasing incidence of vaping cannabinoid-based products, and the constant evolution of product designs, it is important that validated methods with good precision and accuracy be used for analyses of potentially harmful constituents of cannabinoid-based EVP aerosols.

## Figures and Tables

**Table 1:** Instrument Modes and Internal Standard Assignments.

Element Isotope	Instrument Mode	Cell Gas	Quantitated Ion	Quantitated Mass	Internal Standard
^27^Al	MS-MS	H_2_	^27^Al^+^	27	^169^Tm^+^
^52^Cr	MS-MS	NH_3_	^52^Cr(NH3)_2_^+^	86	^103^Rh(NH3)_4_^+^
^56^Fe	MS-MS	NH_3_	^56^Fe(NH3)_2_^+^	90	^103^Rh(NH3)_4_^+^
^59^Co	MS-MS	NH_3_	^59^Co(NH3)_2_^+^	93	^103^Rh(NH3)_4_^+^
^60^Ni	MS-MS	O_2_	^60^NiO^+^	76	^103^RhO^+^
^63^Cu	MS-MS	NH_3_	^63^Cu(NH3)_2_^+^	97	^103^Rh(NH3)_4_^+^
^111^Cd	MS-MS	O_2_	^111^Cd^+^	111	^103^RhO^+^
^118^Sn	SQ	No Gas	^118^Sn^+^	118	^103^Rh^+^
^137^Ba	SQ	No Gas	^137^Ba^+^	137	^169^Tm^+^
^206+207+208^Pb	SQ	No Gas	^206+207+208^Pb^+^	206 + 207 + 208	^103^Rh^+^

Cell parameters: 0.55 mL/min O_2_ cell gas with −20 V octopole bias, −8 V energy discrimination;.4.5 mL/min 10% NH_3_, 90% He cell gas with −18 V octopole bias, −8 V energy discrimination. 5.0 mL/min H_2_ cell gas with −18 V octopole bias, 0 V energy discrimination; No Gas with −8 V octopole bias, 5 V energy discrimination. SQ: Single Quad mode, MS-MS: triple quad mode.

**Table 2:** Accuracy results for spiked aqueous blank.

Analyte	Accuracy Levels	Targets (μg/L)	Average (μg/L)	% RSD	% Accuracy
Al	Low	7.5	7.38	2.9	98
	Mid	25	25.3	2.6	101
	High	40	40.4	3.3	101
Cr	Low	0.375	0.374	3.7	99
	Mid	1.25	1.24	1.5	99
	High	2	1.98	1.1	99
Fe	Low	1.88	1.98	12.1	105
	Mid	6.25	6.16	1.5	99
	High	10	10	4	100
Co	Low	0.375	0.367	1.3	98
	Mid	1.25	1.24	1	99
	High	2	1.97	1.3	99
Ni	Low	0.375	0.379	4.7	91
	Mid	1.25	1.23	1.6	96
	High	2	1.98	2	97
Cu	Low	0.75	0.727	1.8	96
	Mid	2.5	2.43	1.7	97
	High	4	3.86	1.1	96
Cd	Low	0.075	0.073	1.5	97
	Mid	0.25	0.239	2.7	95
	High	0.4	0.385	1.5	96
Sn	Low	0.15	0.145	1.5	96
	Mid	0.5	0.482	1.3	96
	High	0.8	0.772	2	96
Ba	Low	0.375	0.345	5.3	92
	Mid	1.25	1.19	0.9	95
	High	2	1.93	1.2	96
Pb	Low	0.375	0.361	1.4	96
	Mid	1.25	1.21	1.7	96
	High	2	1.93	1.7	97

**Table 3:** Accuracy results for spiked oil matrix.

Analyte	Accuracy Levels	Targets (μg/L)	Average (μg/L)	% RSD	% Accuracy
Al	Low	7.5	7.52	2.1	100
	Mid	25	25.8	2.6	103
	High	40	41.4	2.1	103
Cr	Low	0.375	0.373	0.8	99
	Mid	1.25	1.25	0.8	100
	High	2	2.01	1.2	101
Fe	Low	1.88	1.94	6.8	103
	Mid	6.25	6.17	2.5	99
	High	10	10	1.6	100
Co	Low	0.375	0.372	0.8	99
	Mid	1.25	1.24	1.1	99
	High	2	2.01	1.4	101
Ni	Low	0.375	0.405	9.1	106
	Mid	1.25	1.26	2.2	100
	High	2	2.02	1.8	101
Cu	Low	0.75	0.74	1.5	96
	Mid	2.5	2.4	1.6	97
	High	4	4	1	98
Cd	Low	0.075	0.073	2.7	95
	Mid	0.25	0.246	1.8	98
	High	0.4	0.388	1.9	97
Sn	Low	0.15	0.162	2.1	96
	Mid	0.5	0.502	2.2	97
	High	0.8	0.802	2.8	98
Ba	Low	0.375	0.347	2.5	93
	Mid	1.25	1.21	1	97
	High	2	1.97	1	98
Pb	Low	0.375	0.369	1.8	99
	Mid	1.25	1.23	1.9	98
	High	2	1.99	2.1	99

**Table 4:** Method precision using 8 duplicate runs of quality controls (QCs), µg/L.

	Precision (%RSD) (*n* = 8)				
Analyte	QC Sample	Target	Mean	Repeatability (%)	Intermediate Precision (%)
Al	Low Spike	7.50	7.48	2.7	3.2
	High Spike	40.0	40.9	1.4	2.7
Cr	Low Spike	0.375	0.360	1.8	3.7
	High Spike	2.00	1.95	1.8	3.1
Fe	Low spike	1.88	1.85	2.8	1.1
	High spike	10.0	10.1	1.7	4.0
Co	Low spike	0.375	0.390	2.8	3.7
	High spike	2.00	2.09	2.1	3.8
Ni	Low spike	0.375	0.394	3.5	9.6
	High spike	2.00	2.01	0.9	2.1
Cu	Low spike	0.750	0.744	1.7	2.9
	High spike	4.00	3.97	1.2	2.8
Cd	Low spike	0.0750	0.0725	2.5	2.2
	High spike	0.400	0.394	1.4	2.6
Sn	Low spike	0.150	0.169	2.3	13.7
	High spike	0.800	0.817	1.0	1.1
Ba	Low spike	0.375	0.385	2.1	0.0
	High spike	2.00	2.02	0.5	2.4
Pb	Low spike	0.375	0.368	1.5	0.4
	High spike	2.00	2.01	1.5	0.8

**Table 5:** Reportable level results obtained of EVP samples in ng/10 puffs.

EVPs Samples	Aerosol Mass (mg)	Al	Cr	Fe	Co	Ni	Cu	Cd	Sn	Ba	Pb
LOD		29.3	1.07	107	0.212	3.56	7.50	0.120	2.96	7.30	1.37
Lowest standard		133	6.67	33.3	6.67	6.67	13.3	1.33	2.67	6.67	6.67
Sample 1	32	<LOD	<LOD	<LOD	<LOD	<LOD	73.2	<LOD	4.77	<LOD	<LOD
Sample 2	70	<LOD	<LOD	<LOD	<LOD	15.5	447	<LOD	202	<LOD	<LOD
Sample 3	109	<LOD	<LOD	<LOD	<LOD	<LOD	15.4	<LOD	<LOD	<LOD	<LOD
Sample 4	92	<LOD	<LOD	<LOD	<LOD	36.0	<LOD	<LOD	<LOD	<LOD	6.71
Sample 5	22	<LOD	<LOD	<LOD	<LOD	<LOD	16.1	<LOD	<LOD	<LOD	<LOD

LOD and lowest standard are expressed as ng/10 puffs. Lowest Reportable Level (LRL) is the higher of the concentration of the lowest standard or the LOD in ng/10 puffs.

**Table 6:** Product categorization of the samples by their cannabinoid and nicotine content with concentration range in ng/10 puffs.

Analyte	Nicotine Product				Cannabinoid Product			
	n	Detect *	Mean ± Stdev	Concentration Range	n	Detect *	Mean ± Stdev	Concentration Range
Al	11	0	ND	ND	33	0	ND	ND
Ba	11	0	ND	ND	33	0	ND	ND
Cd	11	0	ND	ND	33	0	ND	ND
Co	11	0	ND	ND	33	0	ND	ND
Cr	11	0	ND	ND	33	0	ND	ND
Cu	11	3	179 (± 235)	[15.4–447]	33	1	16.1	16.1
Fe	11	0	ND	ND	33	0	ND	ND
Ni	11	2	25.8 (± 14.5)	[15.5–36.0]	33	0	ND	ND
Pb	11	1	6.71	[6.71–6.71]	33	0	ND	ND
Sn	11	2	104 (± 140)	[4.77–202.7]	33	0	ND	ND

* 6 samples were positive for both THC and CBD. ND: Not detected.

## Data Availability

Data is available in Accordance with CDC Data Management Plan.
